# How is cognitive behavioural therapy for insomnia delivered to adults with comorbid persistent musculoskeletal pain and disordered sleep? A scoping review

**DOI:** 10.1371/journal.pone.0305931

**Published:** 2024-07-18

**Authors:** Abigail Browne, Roisin Cahalan, Kieran O’ Sullivan

**Affiliations:** 1 School of Allied Health, University of Limerick, Limerick, Ireland; 2 Physical Activity for Health Centre, University of Limerick, Limerick, Ireland; 3 Sports and Human Performance Centre, University of Limerick, Limerick, Ireland; 4 Ageing Research Centre, University of Limerick, Limerick, Ireland; University Hospital Cologne: Uniklinik Koln, GERMANY

## Abstract

**Background:**

Disordered sleep and persistent musculoskeletal pain are highly comorbid. Behavioural interventions such as Cognitive Behavioural Therapy for Insomnia (CBT-I) have shown promise in the management of both disordered sleep and persistent musculoskeletal pain. The aim of this review was to examine how CBT-I is delivered in randomised controlled trials involving people with comorbid disordered sleep and persistent musculoskeletal pain.

**Methods:**

The protocol for this scoping review was registered with the Open Science Framework. Electronic searches of ten database and three clinical trials registries were performed up to 25 October 2023. The methodological quality of each study was evaluated by two independent reviewers using the PEDro tool. The reporting of CBT-I interventions was evaluated using the Template for Intervention Description and Replication (TIDieR) checklist.

**Results:**

Twelve studies met the eligibility criteria. TIDieR scores ranged from 7-11/12, with a mean score of 8.8/12. CBT-I always involved two core components–sleep restriction and stimulus control. Furthermore, an additional five components were usually involved–a cognitive component, sleep hygiene, sleep education, relaxation/deactivation procedures and relapse planning. There was also considerable consistency in the frequency (weekly) and duration (5–9 weeks) of CBT-I programmes. Aspects inconsistently reported included who delivered the intervention; what modifications were made, if any; and the session content and duration. PEDro scores ranged from 5-8/10, with a mean score of 6.7/10.

**Conclusions:**

These findings demonstrate considerable consistency in the components of CBT-I delivered in clinical trials along with the number of sessions. The frequency of sessions was also consistent where almost all studies held weekly session. However, some aspects were either not reported (e.g., precise content of components) or inconsistent (e.g., use of terminology). CBT-I was delivered both individually and in groups. Greater consistency, and more detailed reporting regarding who delivered the intervention, the training provided, and the specific content of CBT-I components would add clarity, and may enhance CBT-I efficacy and allow better replication.

## Introduction

Persistent musculoskeletal (MSK) pain (e.g. low back pain, arthritis) is one of the biggest burdens on healthcare delivery worldwide and a major cause of years lived with health-related disability internationally [1]. Persistent pain is defined as pain that persists for longer than three to six months and is estimated to affect 20% of the world’s population [2]. Insomnia is reported in over half of people with persistent MSK pain [3–5] Disordered sleep and persistent MSK pain have reciprocal effects on one another, and comorbid disordered sleep is recognised as an important issue for clinicians [6, 7]. However, despite emerging evidence of efficacy [8], evidence-based sleep interventions, such as cognitive behavioural therapy for insomnia (CBT-I), are rarely implemented in the clinical management of persistent MSK pain [9], even when disordered sleep is present. Instead, a range of interventions which are more costly, risky and/or less effective (e.g. opioids, surgery, imaging) continue to be offered [10, 11]. A number of guidelines recommend the use of exercise, pacing and other strategies for pain, but none explicitly recommend sleep as a treatment target [12–15].

In recent years psychologically informed rehabilitation approaches have been promoted in the treatment of persistent MSK pain, especially when other comorbid factors (e.g. poor sleep, anxiety, depression) are present [10, 16, 17]. CBT-I appears to be effective for the treatment of both sleep [18] and MSK pain [7] and is more effective for disordered sleep than other non-pharmacological approaches such as sleep hygiene advice [18]. CBT-I is a multi-component treatment for insomnia [19]. Common core components include sleep restriction, sleep hygiene, stimulus control and cognitive therapy [19]. Due to the non-pharmacological nature of CBT-I, it is not associated with the addiction issues surrounding many pharmacological approaches [20]. However, issues such as the training required to deliver CBT-I, and concerns about scope of practice, could limit its uptake and utilisation by clinicians.

Furthermore, while CBT-I is recognised as a first line treatment for insomnia [21], it appears there is considerable variation in how it is delivered across studies [7]. For example, the format could be face to face, online or telephone sessions, and in a group or one-to-one format [22]. The precise content of CBT-I in randomised controlled trials (RCT) appears to vary, including in components such as sleep education, sleep hygiene, relaxation, sleep restriction and cognitive restructuring. Clinicians routinely involved in treating MSK pain (e.g., physiotherapists) have developed skills in psychologically informed care in recent decades [23–25]. However, it is not clear which professionals are involved in the delivery of CBT-I within clinical trials, or how they are trained and supervised.

To better understand how to implement CBT-I for comorbid persistent MSK pain and disordered sleep, a critical evaluation of the delivery of CBT-I in clinical trials is required. Lack of access to psychologists and social workers is a known barrier to implementation of psychologically informed care [9]. This scoping review will inform whether there is potential for first contact practitioners such as physiotherapists, nurses and general practitioners dealing with persistent MSK pain patients, to provide CBT-I. Therefore, this scoping review will explore the delivery of CBT-I in people with comorbid persistent MSK pain and disordered sleep. Specifically, the review will address the following questions:

1. What is the content (e.g., components) of CBT-I delivered to patients with comorbid disordered sleep and persistent musculoskeletal pain in RCTs?
2. What is the format (e.g., delivery mode and personnel, frequency, duration) of CBT-I delivered to patients with comorbid disordered sleep and persistent musculoskeletal pain in RCTs?

## Methods

This scoping review was conducted according to Peters *et al*. (2020) [26] methodology for scoping reviews. There are six steps including: 1) defining the research question/s, 2) identifying relevant studies, 3) study selection, 4) charting the data, 5) collating, summarising, and reporting the results, and 6) consultation. The Preferred Reporting Items for Systematic Reviews and Meta-Analyses (PRISMA) guidelines were followed using the PRISMA extension for scoping reviews (PRISMA-ScR) checklist [27]. An iterative approach was taken towards searching the literature, refining the search strategy, reviewing articles for inclusion, and extracting relevant data.

### Protocol and registration

The scoping review protocol was registered with the Open Science Framework on 02 July 2021. Available at https://osf.io/dvja8/.

### Eligibility criteria

We focused on CBT-I delivered as stand-alone CBT-I or as hybrid CBT-I which included components of CBT-I and CBT- P (Cognitive Behavioural Therapy for Pain) such as CBT-IP or CBT-PI or CBT-C. Due to the high volume of studies available on the topic, and the likelihood of better reporting standards in such studies, we focussed on RCTs exploring CBT-I programmes. No restrictions were placed on the location where care was delivered (e.g., public or private setting or community), the healthcare professional delivering the care, whether it was applied in a group or individual setting, or whether it was delivered in-person or using a telehealth platform.

### Inclusion criteria

1. Studies of adults 18 years and older
2. Studies where all participants reported both comorbid persistent MSK pain (e.g., spinal pain, arthritis, headache) and disordered sleep
3. Completed RCTs, excluding those designated as ‘pilot’ studies.
4. Published in English, or with an English translation

### Exclusion criteria

1. Studies that explicitly included participants with acute pain only (e.g., all participants had pain for <3 months)
2. Studies that explicitly included participants with a diagnosis of sleep disorders such as sleep apnoea, narcolepsy, restless leg syndrome and parasomnias
3. Studies of people with conditions which could clearly cause pain that is not primarily MSK in nature (e.g., cancer, multiple sclerosis etc)
4. Studies of only experimental pain
5. RCTs which were only published as protocol or design papers
6. Secondary analyses of RCTs, where the same data was already published and shortlisted

### Information sources/ search strategy

A comprehensive search strategy was developed with a research librarian. We applied this search strategy to the following nine databases; Medline, EMBASE, CINAHL Complete, PubMed, Academic Search Complete, AMED, APA PsychArticles, APA PsychInfo and SPORTDiscus with Full Text. RCTs from 1996 to 19 August 2021 were initially searched, with a ‘bridge’ search conducted in February 2022 and October 2023. Reference lists of selected articles, meta-analysis and Cochrane reviews were searched for additional studies. Searches were undertaken to find completed studies for registered study protocols and authors of relevant registered study protocols shortlisted were contacted to identify additional studies in print. Grey literature (Open Grey database) and three clinical trial registries (Cochrane Central Register of Controlled Trials (CENTRAL), Clinical Trials Registry (https://clinicaltrials.gov) and Australia and New Zealand Clinical Trials Registry (https://www.anzctr.org.au) were also searched.

### Evidence screen and selection

Removing duplicates of articles was conducted using Rayyan online software [28] https://www.rayyan.ai. Initially, two reviewers selected a sample of eligible titles and abstracts and assessed for suitability. After confirming very good agreement and in line with the AMSTAR checklist (at least 80 percent), the remainder were screened by one reviewer only https://amstar.ca/Amstar_Checklist.php. Where there was insufficient data provided in the title and abstract, the full text was retrieved and analysed to determine eligibility. This process was represented using the Preferred Reporting Items for Systematic Reviews and Meta-Analyses (PRISMA) approach [29].

### Data charting process

Extraction tables were initially piloted with two team members of the team on two articles. Charting was iterative and the form was updated with continuous data extraction.

### Study characteristics

The following data were extracted from each study: Country, Participant Age (mean where available), Sample size, Gender, Type of CBT, Treatment comparison, MSK Condition, and Sleep Criteria.

### Description of CBT-I intervention

The 12 item TIDieR checklist was developed to improve reporting of complex interventions in clinical trials [30]. Items that have no description or are poorly described do not meet the criteria for this item [30]. All data relevant to the TIDieR Checklist were extracted for each study. We have used a TIDieR checklist scoring system developed in a previous study [31] to report if the criteria for each item in the TIDieR Checklist was achieved. For each item, a score of 0 was given if the checklist item was not achieved or partly achieved, a score of 1 was given if the if the checklist item was fully achieved, such that the maximum score was 12. A low scoring study indicated insufficient detail of the intervention was provided and a high scoring study indicated that sufficient detail of the intervention was provided. Each of the studies that did not provide a reference for their treatment manual was emailed asking for a copy of the treatment manual used in their study.

### Critical appraisal of individual sources of evidence

While not considered mandatory in scoping reviews, we felt it was important to critically appraise individual sources of evidence. The Physiotherapy Evidence Base Database (PEDro) is an 11-point scale appraising the methodological quality of RCTs, assessing issues related to bias and quality of reporting [32]. If a PEDro score was already provided on their database (https://pedro.org.au), we used that score. For all other studies, two independent authors assessed the risk of bias and quality of reporting for all studies, and any disagreements were resolved by asking a third author. A score of 1 was given if the study fully achieved the criteria. Total PEDro scores have been interpreted as “fair” (4–5), ‘good’ (6–8) or excellent (9–10) [33].

### Synthesis of results

Descriptive tables and interpretive summaries were produced to report the variables from data extraction.

## Results

Initially, 417 studies were identified from databases (n = 277) and registers (n = 140), while a further 31 studies were found by searching citations and the Open Grey database (see S1 Fig). After removal of duplicates and ineligible studies, a total of 12 studies were eligible for inclusion (see S2 Fig). Key items reported in this review can be located in the PRISMA ScR Checklist (see S3 Fig).

### Study characteristics

The characteristics across the studies are presented in Table 1. Study sample sizes ranged from 28 to 367, with a total of 1316 participants (82.4% female). The mean participant age ranged from 45.0 to 73.1 years. Overall, CBT-I was compared to five active interventions across nine studies (four studies compared CBT-I to cognitive behavioural therapy for pain (CBT-P), two compared to education only, two compared to sleep hygiene, one compared to behavioural desensitisation and one compared to applied relaxation), as well as being compared to usual medical care in three studies, a waiting list control in two studies and a ‘contact control’ condition in one study. CBT-I was delivered either alone (nine studies) or in combination with CBT-P (three studies).

**Table 1:** Characteristics of included studies.

Study	Age	Sample Size	Type of CBT	Comparison	MSK Condition	Sleep Criteria
Currie et al, 2000, [34], Canada	29–59 Average 45.0 (*SD* = 8.0)	n = 60 Male = 27 Female = 33	CBT-I	1. Waiting list control	Chronic pain–confirmed by specialist	Disturbed sleep. Meet criteria for DSM-IV
Edinger et al 2005, [35], USA	21–65 Mean 48.6 (*SD* = 8.2)	n = 47 Male = 2 Female = 45	CBT-I	1. Sleep Hygiene 2. Usual Care	Fibromyalgia–Meet American College of Rheumatology criteria for FM	Meet criteria for DSM-III, have 60 minutes or more of total nocturnal wake time on average over 1 week of sleep log monitoring
Jungquist et al, 2010, [36], USA	25+ Mean 48.7 (*SD* = 10.7)	n = 28 Male = 6 Female = 22	CBT-I	1. Contact control condition	Non-malignant chronic spinal pain–assessed by McGill Pain Index	Insomnia (>30 mins sleep latency +/- minutes wake after sleep onset for >3 days/week for >6 months
Lami et al, 2018, [37], Spain	25–65 Mean 50.19 (*SD* = 8.24)	n = 126 Female = 126	CBT-IP	1. CBT-P 2. Usual Medical Care	Fibromyalgia–Meet American College of Rheumatology criteria for FM	Meet criteria for DSM-IV-TR
Latocha et al, 2023, [38], Denmark	29–75 Mean 60 (*SD* 10)	n = 62 Male = 7 Female = 55	CBT-I	1. Usual Care	Rheumatoid Arthritis—Meet American College of Rheumatology/ European League Against Rheumatism (2010) criteria for RA, with low to moderate disease activity measured with DAS 28-joint count CRP (DAS28-CRP). At the time of screening pharmacological anti-rheumatic treatment had to be unchanged for the preceding 3 months with no planned changes during the trial period.	Meet International Classification of Sleep Disorders–Third Edition duration criterion and Insomnia Severity Index score should be ≥11.
McCrae et al, 2019, [39], USA	18+ Mean 53 (*SD* = 10.9)	n = 113 Male = 3 Female = 110	CBT-I	1. CBT-P 2. Waiting List Control	Fibromyalgia–pain for 6 months and tender point testing—using American College of Rheumatology guidelines	Insomnia (>30 mins sleep latency +/- minutes awake after sleep onset for >3 days/week for >6 months, sleep diary confirmation of insomnia–sleep onset or awake time during the night >30 minutes at east 6 nights during last 2 weeks and daytime dysfunction due to insomnia
McCurry et al, 2021, [7], USA	60+ Mean 70.1 (*SD* = 7.1)	n = 327 Male = 83 Female = 244	CBT-I	1. Education only	Osteoarthritis–a diagnosis of OA in the last 3 years prior to screening	Score of 11+ on Insomnia Severity Index
Prados et al, 2020, [10], Spain	24–62 Mean 49.97 (*SD* = 7.91)	n = 32 Female = 32	CBT-C	1. CBT–P	Fibromyalgia–Meet American College of Rheumatology criteria for FM	Having significant self-reported insomnia according to well established diagnostic criteria, i.e., when the chief complaint is nonrestorative sleep or difficulty in initiating or maintaining sleep and the complaint continues for at least one month (the disturbance must occur at least three times a week for a month).
Smith et al, 2015, [40], USA	50–69 Mean 59.2 (*SD* = 9.9)	n = 100 Male = 21 Female = 79	CBT-I	Behavioural desensitisation	Osteoarthritis–Meet American College of Rheumatology criteria for classification of knee OA	Meet criteria for insomnia disorder and have symptom duration of >1 month, >2 nocturnal awakenings of >15minute duration, or self-reported wake after sleep onset +/- latency to sleep onset of >30 minutes
Vitiello et al, 2013, [41], USA	60+ Mean 73.1 (*SD* = 8.0)	n = 367 Male = 79 Female = 288	CBT-PI	1. CBT-P 2. Education only	Osteoarthritis–Grade II, III or IV pain on the Graded Chronic Pain Scale	Significant insomnia was defined as meeting research diagnostic criteria for insomnia based on self-reported sleep difficulties (trouble falling asleep, difficulty staying asleep, waking too early, or waking up unrefreshed), 3 or more nights per week during the past month with at least one daytime sleep-related problem.
Wiklund et al, 2022, [42] Sweden	21–67 Mean 49.3 (*SD =* 12.3)	n = 54 Male = 9 Female = 45	CBT-I	1. Active Relaxation	Chronic Pain lasting > 3 months	Fulfill the diagnostic criteria for insomnia disorder according to the DSM V and achieve an ISI score of ≥ 14.

SD indicates standard deviation: CBT-I, Cognitive Behavioural Therapy for Insomnia; CBT-IP/CBT-PI, Cognitive Behavioural Therapy for Insomnia and Pain; CBT-C, Cognitive Behavioural Therapy for Insomnia and Pain; CBT P, Cognitive Behavioural Therapy for Pain; MSK, musculoskeletal; FM, fibromyalgia; RA, rheumatology arthritis; OA, osteoarthritis; AVG, Average; BDI, Beck’s Depression Index; DSM–III/IV, IV-TR,V Diagnostic and Statistical Manual of Mental Disorders; DAS, Disease Activity Score; CRP, C-Reactive Protein; DAS28-CRP, Disease Activity Score-28 for Rheumatoid Arthritis with CRP

### Definitions of sleep disorders and pain

Sleep disorders were defined in five different ways (sleep symptom characteristics in five studies, DSM-IV-TR and Insomnia Severity Index in two studies, and DSM-III, DSM-IV and DSM–V in one study each). Five different MSK conditions were included (Fibromyalgia (FM) in five studies, OA in three studies, chronic pain in two studies and rheumatoid arthritis and spinal pain in one study each). Two definitions for FM were used across the five FM studies, with four studies using the American College of Rheumatology (ACR) criteria [43] and the remaining study using the ACR guidelines (pain for six months along with positive tender point testing). Definitions for OA varied in three ways (three-year diagnosis of OA prior to screening in one study, ACR diagnosis for OA knee in one study [44] and grade II, III or IV pain on the graded chronic pain scale in one study).

### Reporting

#### TIDieR

TIDieR scores ranged from 7-11/12, with a mean of 8.8/12 (see Table 2). All studies met the criteria for items 1, 2, 3, 4, and 6, such that the content (what materials, what procedures) and aspects of the format (when) were well described. Consistently low-scoring items of the TIDieR checklist were items 5 (who provided the intervention) where four studies met the criteria [7, 36, 39, 41], item 12 (intervention delivered as planned) where three studies met the criteria [7, 39, 40] and item 10 (modifications made), where no study met the criteria. The data from individual sources of evidence are charted in the TIDieR Checklist (see Table 3).

**Table 2:** TIDieR checklist scores.

Study	Currie et al, 2000 [34]	Edinger et al, 2005 [35]	Jungquist et al, 2010 [36]	Lami et al, 2018 [37]	Latocha et al, 2023 [38]	Martínez et al, 2014 [18]	McCrae et al, 2019 [39]	McCurry et al, 2021 [7]	Prados et al, 2020 [10]	Smith et al, 2015 [40]	Vitiello et al, 2013 [41]	Wiklund et al, 2022 [42]
**Brief Name– 1**	1	1	1	1	1	1	1	1	1	1	1	1
**Why– 2**	1	1	1	1	1	1	1	1	1	1	1	1
**What Materials– 3**	1	1	1	1	1	1	1	1	1	1	1	1
**What Procedure– 4**	1	1	1	1	1	1	1	1	1	1	1	1
**Who Provided– 5**	0	0	1	0	0	0	1	1	0	0	1	0
**How– 6**	1	1	1	1	1	1	1	1	1	1	1	1
**Where– 7**	0	0	0	1	1	1	1	1	1	1	1	1
**When and How Much– 8**	1	1	1	1	1	1	1	1	1	1	1	0
**Tailoring– 9**	1	1	1	0	0	0	1	1	1	1	0	1
**Modifications– 10**	0	0	0	0	0	0	0	0	0	0	0	0
**How Well–Planned– 11**	1	0	1	1	1	1	1	1	1	1	1	1
**How Well–Actual– 12**	0	0	0	0	0	0	1	1	0	1	0	0
**Total**	**8**	**7**	**9**	**8**	**8**	**8**	**11**	**11**	**9**	**10**	**9**	**8**

Marking: 1 = criteria fully achieved, 0 = criteria not achieved or partly achieved

**Table 3:** TIDieR checklist.

TIDieR Item	(Currie *et al*. 2000) [34]	(Edinger *et al*. 2005) [35]
**Brief Name (1) **	CBT-I secondary to chronic pain	CBT-I for fibromyalgia patients
**Why (2) **	RCT to investigate the efficacy of the intervention in patients with insomnia secondary to chronic pain	RCT to investigate if CBT-I would produce greater improvements in sleep, pain, mood, and mental wellbeing than sleep hygiene and usual care
**What Materials (3) **	Written information provided to patients–details of unpublished manuscript provided but unable to locate. Therapists followed a structured written manual to ensure consistency–no direction where to source.	Written information provided (pamphlet) to patients and they listened to a standardised audio cassette in session one–no direction on where to source provided. A study treatment manual was used by therapists.
**What Procedures (4) **	CBT-I: Sleep restriction Stimulus control Cognitive restructuring Sleep education Sleep hygiene Relaxation training Waiting List Control: Sleep diary for 7 weeks Weekly 10 min phone call to encourage adherence	CBT-I: Stimulus control Sleep restriction Cognitive therapy Sleep education SH: Sleep education Stimulus control Sleep hygiene Usual Care: No behavioural therapy Completed a sleep log/actigraphy data/questionnaires
**Who provided (5) **	Six doctoral students or interns in Clinical psychology. Provider expertise not recorded. States previous training in CBT interventions but no further details provided	Two male clinical psychologists. One therapist has 6 years’ experience and the other had 18 years’ experience treating patients with sleep disorders. No specific training recorded
**How–delivery mode (6) **	Five face to face groups of 5–7 participants	Face to face in six weekly individual sessions
**Where–location (7) **	N/R	N/R
**When and how much (8) **	Seven 2-hour weekly sessions	CBT-I and SH had first weekly session of 45–60 minutes, subsequent sessions 15–30 minutes.
**Tailoring (9) **	Sleep restriction was tailored at each session.	TIB prescription was provided for all CBT-I participants, and this was tailored throughout the study.
**Modifications (10)**	N/R	N/R
**How well–planned (11)**	Fidelity–each session was led by a primary therapist and a cotherapist. Therapists followed a structured written manual to ensure treatment components were applied consistently across therapy groups. Weekly therapist supervision on a session-by-session basis to review the process of the sessions, ensure therapist adherence to the treatment manual and troubleshoot around specific clinical issues.	N/R
**How well–actual (12) **	N/R	N/R
**TIDieR Item **	**(Jungquist *et al*. 2010) [36]**	**(Lami *et al*. 2018) [37]**
**Brief Name (1) **	CBT-I for insomnia in chronic pain	Efficacy of CBT-I for Insomnia and pain in patients with fibromyalgia
**Why (2) **	Garner additional evidence regarding CBT-I efficacy in treating insomnia in chronic pain context and to assess whether it is associated with clinical changes with respect to pain severity +/-interference.	RCT to identify the clinical benefits of CBT-IP compared to CBT-P and UMC in FM.
**What Materials (3) **	Written materials posted to each participant before the first session. Reference provided for the published treatment manual.	Written information provided to participants in the form of a therapy manual containing the necessary information and tasks involved in each session. No reference was provided to this manual.
**What Procedures (4) **	CBT-I: Sleep restriction Stimulus Control Cognitive restructuring Sleep hygiene Relapse prevention Control: Discuss sleep/pain diary and BDI	CBT-IP: W1 –Education about FM and sleep and psychoeducation about sleep problems and insomnia W2 –Sleep hygiene W3 –Sleep restriction and stimulus control W4—Relaxation W5 –Planning activities—pacing W6 –Communication and relationship with others W7 –Cognitive therapy for pain and sleep W8 –Cognitive restructuring W9 –Integration of treatment components, relapse management, maintenance of goals CBT-P: W1 –Education about FM and pain and psychoeducation about pain W2 –Relaxation W3 –Education and training on emotions and pain and fear of pain W4 –Planning activities–pacing W5 –Communication and relationship with others W6 –Training in problem solving W7 –Cognitive therapy for pain W8 –Cognitive restructuring W9 –Integration of treatment components, relapse management, maintenance of goals Usual Medical Care
**Who provided (5) **	Masters trained nurse therapist who attended a 2.5-day seminar on diagnosing and treating sleep disorders and the delivery of CBT-I, viewed a series of videotaped CBT-I sessions conducted by a licenced clinical psychologist who had experience in conducting CBT-I in research and clinical venues. They received weekly supervision (60–120 minutes per week) for the duration of the study by experienced therapist.	No provider background is recorded and “therapists” is stated without describing what kind. Therapists with high level of training, and experience in the domain of chronic pain and sleep disorders No specific training recorded
**How–delivery mode (6) **	Eight individual face to face sessions (although not explicitly stated individual)	Nine face to face group sessions (5/7 participants)
**Where–location (7) **	N/R	Psychology clinic of University of Grenada, Spain
**When and how much (8) **	CBT-I—Eight weekly sessions of between 30–90 minutes	Nine weekly 90 minute sessions
**Tailoring (9) **	TIB set in initial weeks and progressed throughout the study	N/R
**Modifications (10) **	N/R	N/R
**How well–planned (11)**	Therapists attended weekly supervision sessions for 60–120 minutes for the duration of the study by experienced therapist	There were regular clinical meetings between therapists and the research group, and video recordings to monitor the implementation of the intervention
**How well–actual (12) **	N/R	N/R
**TIDieR Item **	**(Latocha et al 2023) [38]**	**(Martínez et al. 2014)[18] **
**Brief Name (1) **	CBT-I for RA	CBT-I and sleep hygiene in FM
**Why (2) **	To compare the immediate effect of CBT-I, relative to usual care, on sleep efficiency at week 7 in patients with RA	Collect evidence of the efficacy of CBT-I to treat insomnia in FM
**What Materials (3) **	A treatment manual was used–no reference supplied. Emailed corresponding author for manual	Written therapy manual with detailed information about each session was used–no reference provided.
**What Procedures (4) **	CBT-I W1 –Introduction, sleep education and sleep restriction W2 –Sleep education, sleep hygiene, stimulus Control, sleep restriction and relaxation W3 –Stimulus control, sleep restriction and cognitive therapy W4 –Sleep restriction, cognitive therapy and relaxation W5 –sleep restriction, recap and relapse prevention W6 –Sleep restriction, recap and closure Usual Care	CBT-I: W1 –Sleep education and sleep hygiene W2 –Sleep restriction and stimulus control W3 –Psychological deactivation procedures W4 –Cognitive therapy to change negative thoughts about insomnia W5– Cognitive therapy to change negative thoughts about insomnia W6 –Maintaining achievements/managing relapses SH: W1 –Sleep education and sleep hygiene W2 –SH rules related to environmental factors W3 –Lifestyle factors W4 –Diet W5 –Physical Exercise W6 –Maintaining achievements/managing relapses
**Who provided (5) **	One registered nurse with no provider expertise recorded who was trained in the CBT-I masterclass at University of Oxford and the CBT-I Advanced Course at the University of Pennsylvania	Three female therapist’s—no background provided. Therapists experienced in chronic pain and sleep issues. No specific training recorded.
**How–delivery mode (6) **	Group face to face sessions	Face to face in groups of 5/6
**Where–location (7) **	Centre for Rheumatology and Spine Diseases, Rigshospitalet Glostrup, Denmark	Psychology Clinic of University of Grenada, Spain
**When and how much (8) **	Six two-hour long weekly sessions.	1.5-hour sessions, once a week for 6 weeks
**Tailoring (9) **	Sleep restriction addressed throughout the study, but not detailed	TIB restrictions established, and the sleep schedule was reviewed every 3–4 days by increasing bedtime as sleep efficiency was improving.
**Modifications (10) **	N/R	N/R
**How well–planned (11) **	Fidelity–Before recruitment to the trial was initiated, the nurse had several consultations with patients with RA reporting insomnia in the outpatient clinic to enhance her CBT-I experience. These consultations were supervised by a psychologist, and feedback was given to improve the nurse’s CBT-I skills. To ensure that the intervention was delivered as intended by the manual, Microsoft PowerPoint and worksheets were used throughout the intervention to aid delivery. The intervention manual was developed in collaboration with two patient partners and a clinical psychologist	Fidelity–therapists had high level of training an experience, a written therapy manual was used, and therapists had regular clinical meetings with the research group to monitor the implementation of the therapies and follow the progress of patients.
**How well–actual (12) **	N/R	N/R
**TIDieR Item**	**(McCrae *et al*. 2019) [39]**	**(McCurry *et al*. 2021) [7]**
**Brief Name (1) **	CBT-I and pain in adults with comorbid chronic insomnia and FM–SPIN study	Effect of Telephone CBT-I in older OA pain
**Why (2) **	Compares the use of CBT-I, CBT-P, and a WLC condition of sleep and pain-related outcomes patients with FM	Evaluate the effectiveness of telephone CBT-I compared with attention control in moderate to severe OA pain patients and insomnia.
**What Materials (3) **	A workbook was provided to participants detailing treatment instructions and rationale. Protocols were manualised. No reference provided or attachment to published article. A passive relaxation audiotape was provided to participants to practice every day.	Written materials posted to each participant before first session. No reference provided to source the materials however, manuals and handouts provided on request..
**What Procedures (4) **	CBT-I: W1 –Sleep education W2 –Sleep hygiene and stimulus control W3 –Relaxation W4 –Sleep restriction W5– Cognitive therapy–monitoring automatic thoughts W6 –Cognitive therapy–challenging/replacing dysfunctional thoughts W7 –Cognitive therapy–Practical recommendations W8—Review of skills and long-term maintenance CBT-P: W1 –Pain education and diaphragmatic breathing W2 –Passive muscle relaxation W3 –Activity-rest cycle and autogenic relaxation W4 –Visual Imagery W5– Cognitive therapy–monitoring automatic thoughts W6 –Cognitive therapy–challenging/replacing dysfunctional thoughts W7 –Cognitive therapy–balanced thinking W8—Review of skills and long-term maintenance Waiting List Control	CBT-I: W1 –Information on insomnia and sleep Behavioural/scheduling: Stimulus control W2 –Behavioural/scheduling: Bed (sleep) restriction W3 –Sleep education W4 –Constructive worry Relaxation/mindfulness W5 –Sleep hygiene recommendations Adopting beliefs/attitudes that foster good sleep W6 –Review of behavioural and cognitive strategies Maintaining treatment gains / relapse prevention Education: W1 –Sleep education and sleep hygiene W2 –Sleep and OA education W3 –Sleep and pain education W4 –Sleep and OA education W5 –Sleep and OA education W6 –Sleep and pain education
**Who provided (5) **	Predoctoral students in clinical psychology whose training involved mock therapy with corrective feedback and audiotaped practice sessions with volunteers	Masters level psychologist and PhD level nurse and social worker. All interventionists received one day training for each intervention condition led by experts in CBT-I and OA pain education
**How–delivery mode (6) **	Face to face individual sessions	Individual telephone CBT-I sessions which were recorded
**Where–location (7) **	University of Florida, USA–no specific location detail provided	At patients home
**When and how much (8) **	50-minute sessions, weekly for 8 weeks	Six 20/30-minute sessions over eight weeks
**Tailoring (9) **	TIB tailored to each patient and the interventionist and participant worked together to set regular bed/wake times to help the participant follow the prescription. Patients were questioned during sessions about their home practice of techniques and procedural modification were adopted as needed.	TIB calculated for all and progressed throughout the duration of the study if improvements produced
**Modifications (10) **	N/R	N/R
**How well–planned (11) **	Fidelity—treatments developed by psychologists, they provided training, weekly supervision, and ongoing monitoring of treatment delivery via audiotape. Half of the sessions were randomly selected for scoring by another interventionalist and 25% of the scored tapes were double scored by the leading supervising clinical psychologist. interventionist scoring of each other’s tapes (not their own) was used because they were highly qualified to evaluate session content. Also, viewing others’ tapes provided valuable booster training and enhanced consistency across interventionists. Session parts were weighted by importance and scored 0, 0.5, or 1 and for no, part, or full delivery, respectively. Scores were summed to provide and index of the degree of treatment delivery. A separate index of treatment purity was calculated using similar weighted scoring procedure. Participants also completed a 10-item quiz on treatment rationale and procedures at the beginning of session 3 and a treatment credibility questionnaire at the end of session 3. Interventionists left the room prior to the completion of the credibility questionnaire, which was then completed by the participants, placed in a sealed envelope, and given to the project coordinator.	Fidelity–Training included review by a co-author of all 6 recordings for 3 pilot cases (2 CBT-I and 1 EOC). Thereafter, 2 sessions for each participant (1 randomly selected and 1 chosen by either the coach or reviewer) were reviewed to maintain treatment fidelity and ensure no cross-treatment contamination. Fidelity reviews were discussed during weekly team meetings.
**How well–actual (12) **	Average compliance rates for sleep hygiene, stimulus control, and relaxation components of CBT-I were 91% during treatment, 86% at posttreatment, and 85% at follow up. Likewise, the average compliance rates for the relaxation and activity pacing components of CBT-P were 90% during treatment, 90% at posttreatment, and 62% at follow up. Average total credibility scores for CBT-I (M = 8.93, SD = 1.21) and CBT-P (M = 8.38, SD– 1.37) did not significantly differ.	Study retention was 83% and 88% at 2 months and 73% and 78% at 12 months for CBT-I vs EOC, respectively. Most participants completed all 6 CBT-I (130 of 163[80.0%]) or EOC (147 of 164[89.6%]) sessions. The MS level coach delivered the most treatment sessions (267 of 327[81.6%]). There were no significant differences between treatment conditions in number of telephone sessions attended. Session length (median, 23.4 and 23.0 minutes for CBT-I and EOC, respectively), or distribution of CBT-I vs EOC participants among coaches.
**TIDieR Item **	**(Prados *et al*. 2020) [10]**	**(Smith *et al*. 2015) [40]**
**Brief Name (1) **	CBT-I for FM–effects on polysomnography parameters and perceived sleep quality	CBT-I in knee OA
**Why (2) **	Compare the efficacy of CBT-P and CBT-C with regards to sleep quality on FM patients suffering from insomnia	Evaluate the efficacy of CBT-I, to determine whether improvements in sleep are associated with improvements in clinical pain, and to determine whether alterations in laboratory measures of pain modulation mediate sleep-related improvements in clinical pain
**What Materials (3) **	A specific manual for each therapy modality was used that included detailed information about each session–no reference was provided	A published treatment manual was used–reference supplied
**What Procedures (4) **	CBT-C: W1 –Psychoeducation about FM and pain psychophysiology and basic sleep education W2 –Sleep hygiene W3 –Sleep restriction and stimulus control W4 –Training in physiological deactivation procedures W5 –Activity pacing and scheduling W6 –Communication skills training W7 –Cognitive Therapy–sleep and pain W8 –Cognitive Therapy–sleep and pain W9 –Summary and Maintaining achievements/managing relapses CBT-P: W1 –Psychoeducation about FM and pain psychophysiology W2 –Training in physiological deactivation procedures W3 –Emotion management W4 –Activity pacing and scheduling W5 –Communication skills training W6 –Problem-solving strategies W7 –Cognitive Therapy–pain W8 –Cognitive Therapy–pain W9 –Summary and Maintaining achievements/Managing relapses	CBT-I: Sleep restriction Stimulus control Cognitive therapy for insomnia Sleep hygiene education Behavioural Desensitisation
**Who provided (5) **	Three postdoctoral level researchers in clinical psychology with expertise in CBT tailored to patients suffering from sleep disturbances and chronic pain conditions. No specific training recorded.	Seven post-doctoral clinical psychology fellows or faculty and two advanced doctoral candidates with experience in behavioural medicine and two doctoral candidates. No specific training recorded.
**How–delivery mode (6) **	Face to face in groups of 5/7	Individual face to face sessions described in treatment manual not in the paper itself.
**Where–location (7) **	Clinical Psychology Unit of University of Grenada, Spain	John Hopkins Bayview Medical Centre, Baltimore, USA
**When and how much (8) **	1.5-hour sessions, once a week for 9 weeks	Eight 45-minute sessions.
**Tailoring (9) **	TIB calculated for all participants, and each was supervised with progression of sleep restriction schedules	Sleep restriction calculated using TST and a PTIB was issued for each participant. This was altered throughout the course of the study.
**Modifications (10) **	N/R	N/R
**How well–planned (11) **	Fidelity–One therapist in charge of unique group in each treatment condition, the same applies for the two other therapists. This was to control inherent personal features of each therapist. A specific manual was used which had been created, designed, and piloted by the three researchers. There were regular clinical meetings between the researchers and the research group, and video recordings to monitor implementation of the intervention.	Fidelity–one author supervised the interventionists weekly. Interventionists completed a structured checklist of session elements. Audiotapes of all sessions and randomly selected 15% of records from each interventionalist had rated them for the presence or absence of session-specific intervention elements. Independent auditors also rated the sessions for contamination. Adherence–Questionnaires were administered before each treatment session asking patients to rate how closely and frequently, they followed their interventionists various prescriptions in the preceding week: ratings were made on a Likert scale, where 0 = not at all and 7 = followed precisely every day. The participants were informed that the interventionists were blinded to the responses of the questionnaires.
**How well–actual (12) **	N/R	The mean percentages of treatment elements rated as present in the randomly selected sample of session recordings were 91% for behavioural desensitisation and 97% for CBT-I (*P* = 0.118). Furthermore, none of the sessions were rated as contaminated by elements from the other treatment. At the end of treatment, there was no observed between-group differences in self-reported adherence to the interventionists behavioural prescriptions at any treatment session. Overall adherence ratings were mean 5.49 (SD 0.93) for CBT-I and mean 5.50 (SD1.29) for behavioural desensitisation (*P* = 0.97).
**TIDieR Item **	**(Vitiello *et al*. 2013) [41]**	**(Wiklund *et al* 2022) [42]**
**Brief Name (1) **	CBT-I for OA pain in Primary Care	CBT-I comorbid with chronic pain
**Why (2) **	To assess if integrated group-format cognitive-behavioural intervention for chronic pain and insomnia and a group cognitive behavioural pain coping skills intervention differ in efficacy for sleep and pain outcomes from a group education-only control intervention. The study also assessed if an integrated group behavioural intervention for chronic pain and insomnia differs in efficacy for sleep and pain from a group cognitive-behavioural pain coping skills intervention.	To investigate the acceptability of ability of an ICBT-I treatment and assess whether ICBT-I is more effective than internet- administered applied relaxation in reducing insomnia symptoms comorbid to chronic pain
**What Materials (3) **	A comprehensive treatment manual was used–no reference provided.	Reference to manual for active relaxation. Interventions were delivered online but unclear if live
**What Procedures (4) **	CBT-PI: Cognitive restructuring Stimulus control Sleep restriction Sleep Hygiene education Pain and sleep education Physical activation Goal setting Relaxation Activity pacing Guided imagery Daily sleep monitoring CBT-P: Pain education Physical activation Goal setting Relaxation Activity pacing Guided imagery Cognitive restructuring EOC: Educational content related to pain and sleep management	CBT-I W1 –Rationale and sleep diary W2 –Sleep restriction W3 –Stimulus control W4 –Activity balance W5 –Relapse prevention Applied Relaxation: W1 –Rationale, sleep diary, progressive relaxation and diaphragmatic breathing W2 –not documented W3 –conditioned relaxation W4 –differentiated relaxation W5 –quick relaxation
**Who provided (5) **	One Masters level family counsellor and one PhD psychologist both experienced in working with older adults but neither had prior experience with cognitive behavioural interventions for insomnia or osteoarthritis pain. Interventionists received 6 weeks of training by clinical psychologist co-investigators with substantial expertise and experience in protocol-based cognitive-behavioral interventions for insomnia and osteoarthritis pain.	Masters students in psychology or senior psychologists supervised by senior psychologists trained in CBT-I
**How–delivery mode (6) **	Face to face in groups of 5–12 (9.4 average)	Face to face in groups of 5–12 (9.4 average)
**Where–location (7) **	In a classroom setting at primary care clinics, Washington, USA	Online
**When and how much (8) **	Six-weekly 90-minute group sessions	Five weekly sessions of unknown duration
**Tailoring (9) **	Sleep restriction occurred but no description provided on how this was calculated and titrated.	Sleep restriction prescription was equal to the average total sleep time for each participant. This was altered throughout the course of the study.
**Modifications (10) **	N/R	N/R
**How well–planned (11) **	Fidelity–one author supervised the interventionists weekly. Interventionists completed a structured checklist of session elements. Audiotapes of all sessions and randomly selected 15% of records from each interventionalist had rated them for the presence or absence of session-specific intervention elements. Independent auditors also rated the sessions for contamination. Adherence–Questionnaires were administered before each treatment session asking patients to rate how closely and frequently, they followed their interventionists various prescriptions in the preceding week: ratings were made on a Likert scale, where 0 = not at all and 7 = followed precisely every day. The participants were informed that the interventionists were blinded to the responses of the questionnaires.	Fidelity–Both treatment arms had therapist support every week of treatment. Support was provided via written messages in the treatment platform. Therapists (master’s students in psychology or senior psychologists) provided problem solving and feedback on weekly tasks and ensured the correct implementation of treatment components. Because therapist support is one of the factors that contribute to treatment outcome [30], there was no restriction of therapist support, as it did not include components from the other treatment arm. The master’s students were supervised by senior psychologists trained in CBT (TW and PM). In a few cases, telephone calls were used to solve technical problems or to reach participants who did not respond to written messages. The same therapists provided both treatments, and the participants were distributed because of the randomization. Because the treatment content was standardized in both treatment arms, no measures of therapist fidelity were collected.
**How well–actual (12) **	Key treatment elements were noted as present for both behavioural desensitisation (91%) and CBT-I (97%) in the sampled recordings. *P* = 0.118). No contamination of treatment sessions was noted. No between-group differences in self-reported adherence between groups. Overall adherence ratings were mean 5.49 (SD 0.93) for CBT-I and mean 5.50 (SD1.29) for behavioural desensitisation (*P* = 0.97).	N/R

CBT indicates Cognitive Behavioural Therapy, CBT-I, Cognitive Behavioural Therapy for Insomnia; CBT-IP/CBT-PI, CBT-C Cognitive Behavioural Therapy for Insomnia and Pain; CBT P, Cognitive Behavioural Therapy for Pain; ICBT-I, internet-delivered Cognitive behavioural therapy for insomnia; RCT, randomised controlled trial; SH, sleep hygiene; N/R, not reported; UC, usual care; TIB, time in bed; UMC, usual medical acre; FM, fibromyalgia; BDI, Beck’s Depression Index; W1, week one; SPIN, Sleep and Pain Interventions for patients with FM; WLC, waiting list control; OA, osteoarthritis; RA, Rheumatoid Arthritis; MS, Master of Science; PhD, doctor of philosophy; EOC, education only control, TST, total sleep time; PTIB, prescribed time in bed; M, mean; SD, standard deviation

#### PEDro

PEDro scores were available on the database for four studies [7, 18, 34, 45], and were calculated for the remaining eight studies [10, 35–40, 42] (see Table 4). PEDro scores ranged from 5-8/10, with a mean of 6.7/10. Ten studies were deemed good quality, with two deemed fair quality [34, 42]. All 12 studies met the criteria for random allocation, between group comparison and reporting both point estimates and variability. Ten studies had baseline comparability [7, 10, 18, 34–37, 39–41] and analysed data using intention to treat principles [7, 10, 34–36, 38–42]. As is typical of interventions such as CBT-I, no study had blinded clinicians delivering the intervention, but two studies report blinded participants [7, 41]. Eight studies had blinded assessors [7, 10, 18, 37–41]. Six studies documented concealed allocation [10, 37–39, 42, 46].

**Table 4:** PEDro scores.

Study	Currie et al, 2000 [34]	Edinger et al, 2005 [35]	Jungquist et al, 2010 [36]	Lami et al, 2018 [37]	Latocha et al, 2023 [38]	Martínez et al, 2014 [18]	McCrae et al, 2019 [39]	McCurry et al, 2021 [7]	Prados et al, 2020 [10]	Smith et al, 2015 [40]	Vitiello et al, 2013 [41]	Wiklund et al, 2022 [42]
**Eligibility Criteria**	Yes	Yes	Yes	Yes	Yes	Yes	Yes	Yes	Yes	Yes	Yes	Yes
**Randomly Allocation**	1	1	1	1	1	1	1	1	1	1	1	1
**Concealed Allocation**	0	0	0	1	1	1	1	0	1	0	0	1
**Baseline Comparability**	1	1	1	1	0	1	1	1	1	1	1	0
**Blind Subjects**	0	0	0	0	0	0	0	1	0	0	1	0
**Blind Therapists**	0	0	0	0	0	0	0	0	0	0	0	0
**Blind Assessors**	0	0	0	1	1	1	1	1	1	1	1	0
**Adequate Follow-up**	0	1	0	0	1	1	1	1	0	1	1	0
**Intention to Treat**	1	1	1	0	1	0	1	1	1	1	1	1
**Between-Group Comparisons**	1	1	1	1	1	1	1	1	1	1	1	1
**Point Estimates and Variability**	1	1	1	1	1	1	1	1	1	1	1	1
**Total **	5*	**6 **	**6 **	**6 **	**7**	7*	**8 **	8*	**7 **	**7 **	8*	**5**

Marking: 1 = included, 0 = not included, ^#^Criteria 1 does not contribute to total score. * score provided by PEDro on www.pedro.org.au

#### CBT-I content

Table 5 illustrates the specific components of CBT-I identified by each study. Two components—sleep restriction and stimulus control—were included in all 12 studies. No details were provided across the studies to indicate adherence to sleep restriction. Four other components were usually involved in trials of CBT-I. Eleven studies included a cognitive component, with one study excluding it to try to keep the intervention as brief as possible [42]. Ten studies included sleep hygiene [7, 10, 18, 34, 36–41] and nine studies included sleep education [7, 10, 18, 34, 35, 37–39, 41], and relaxation or psychological/physiological deactivation procedures [7, 10, 18, 34, 37–39, 41, 42]. Eight studies Included relapse planning [7, 10, 18, 36–39, 42].

**Table 5:** CBT-I Components present in each study.

Component	Currie et al, 2000 [34]	Edinger et al, 2005 [35]	Jungquist et al, 2010 [36]	Lami et al, 2018 [37]	Latocha et al, 2023 [38]	Martínez et al, 2014 [18]	McCrae et al, 2019 [39]	McCurry et al, 2021 [7]	Prados et al, 2020 [10]	Smith et al, 2015 [40]	Vitiello et al, 2013 [41]	Wiklund et al, 2022 [42]
**Sleep Restriction– 12**	x	x	x	x	x	x	x	x	x	x	x	x
**Stimulus Control– 12**	x	x	x	x	x	x	x	x	x	x	x	x
**Cognitive Component– 11**	x	x	x	x	x	x	x	x	x	x	x	-
**Sleep Hygiene– 10**	x	-	x	x	x	x	x	x	x	x	x	-
**Sleep Education– 9**	x	x	-	x	x	x	x	x	x	-	x	-
**Relaxation/psychological/physiological deactivation procedures– 9**	x	-	-	x	x	x	x	x	x	-	x	x
**Relapse Planning– 8**	-	-	x	x	x	x	x	x	x	-	-	x

Marking: x = component included in study,— = component not included in study

All studies provided physical or information materials to the participants or the interventionists. There was variation in what materials were used. Two studies provided detailed treatment manuals and referenced where to access them [36, 40]. Three studies state they provided written information, but failed to describe the depth of content and a reference to where to locate this information [7, 34, 38]. The remaining six studies stated they provide a detailed therapy manual but failed to reference where to access them [10, 18, 35, 37, 39, 41]. One study described providing standardised care, but does not describe using a manual [42]. Each of the ten studies that failed to provide a reference for the treatment manual were sent an email. At the time of publication, one author provided a copy of their treatment manual [7]. Three studies failed to describe where the intervention was delivered [34–36], however there was very little detail provided across the remaining studies on this item. No studies describe the process of content design nor did any study describe using public and patient involvement.

#### Component content variation

Variation was found in the content of each component of CBT-I. Most studies provided detail on the content of each component, however some studies provided limited or no detail [10, 18, 37, 38, 41, 42]. This impacted on our ability to fully map the content of CBT-I. Monitoring and adjusting time in bed (TIB) was a frequently adapted strategy within Sleep Restriction, but there was much variation in prescription of same (see Table 6). Similarly, four studies provided no detail on what content was delivered on stimulus control [10, 18, 38, 41] and one study stated that they “reinforced the relationship between sleep and bed” without any further details [37]. Of the remaining studies, there was much agreement on the advice provided, but considerable variation on some aspects, such as avoiding napping (see Table 7).

**Table 6:** Sleep restriction protocols.

Study	TIB Prescription	Sleep Window	Sleep Increment	Progress/ Regress
Currie et al, 2000, [34]	Participants were instructed to reduce their TIB to the TST recorded during the baseline self-monitoring period. In subsequent weeks, each participant was permitted to increase his or her sleep window gradually until SE stabilised at approximately 85%. The application of these rules was flexible and tailored to the individuals sleep schedule and acceptance of the procedure. In most cases participants delayed their bedtime in order to comply with the sleep window, whereas some participants opted to get up earlier in the morning to shorten their bed time. with the goal of achieving 85% SE within 4–5 weeks.	TST = TIB Never less than 4 hours	15–30 minutes per week	No specific guidance
Edinger et al 2005, [35]	An initial TIB prescription was set at the average baseline log sleep time plus 30 minutes was provided to each CBT patient. Remaining sessions entailed reviewing instructions and adjusting TIB.	Average baseline log sleep time plus 30 minutes	15 minutes	SE of 85% or greater and noted daytime sleepiness, increase by 15 minutes SE of less than 80%, decrease by 15 minutes Otherwise TIB held constant
Jungquist et al, 2010, [36]	(1) establish a fixed wake time and (2) decrease sleep opportunity by limiting the subject’s TIB to an amount that equals their average TST as ascertained by baseline sleep diary measures. Once a target amount of time in bed is set, the patient’s bedtime is delayed to later in the night so that the TIB and average TST are the same.	TST = TIB No less than 4.5 hours	15 minutes	SE 90% or more
Lami et al, 2018, [37]	N/R			
Latocha et al, 2023, [38]	N/R	N/R	N/R	N/R
Martínez et al, 2014, [18]	Participants were given instructions for applying sleep restriction and were asked to complete a sleep diary before applying sleep restriction. Sleep restriction was calculated considering the sleep diary data, restriction of time in bed was established, bedtime and time to get up were set, and the sleep schedule was reviewed every 3–4 days by increasing bedtime as sleep efficiency was improving.	N/R	N/R	N/R
McCrae et al, 2019, [39]	A time in bed prescription was determined by adding 30 minutes to the participants’ baseline average total sleep time. The interventionist and the participant worked together to set regular/bed wake times to help the participant follow the prescription.	TST + 30 minutes No less than 5 hours	N/R	N/R
McCurry et al, 2021, [7]	Participants were given an in-bed restriction plan that initially matched the average sleep time reported in baseline diaries.	TST = TIB No less than 6 hours	15 Minutes	SE 85% or greater
Prados et al, 2020, [10]	Sleep diary data used to calculate SE and implement a personal sleep schedule for sleep restriction. Participants were trained to review their sleep schedule twice a week to assess their SE and were supervised by the therapist in a weekly session.	N/R	N/R	N/R
Smith et al, 2015	(1) establish a fixed wake time and (2) decrease sleep opportunity by limiting the subject’s TIB to an amount that equals their average TST as ascertained by baseline sleep diary measures. Once a target amount of time in bed is set, the patient’s bedtime is delayed to later in the night so that the TIB and average TST are the same.	TST = TIB No less than 4.5 hours	15 minutes	SE 90% or more
Vitiello et al, 2013	N/R	N/R	N/R	N/R
Wiklund et al, 2022, [42]	On the basis of the sleep diary data, an individually designed sleep prescription (bedtime) was calculated and applied during the second week.	Average TST. No less than 4 hours	15–30 minutes	SE over 85%/under 75%

Abbreviations: CBT, Cognitive behavioural therapy; N/R, not reported; SE, sleep efficiency; TIB, time in bed; TST, total sleep time

**Table 7:** Stimulus control protocols.

Study	Description
Currie et al, 2000, [34]	“Strengthen the association between their beds and sleeping”. Avoid napping, go to bed only when sleepy, to use the bedroom for sleep and sex, establish a pre-sleep routine to be used every night and get out of bed if unable to fall asleep within 20 minutes. Maintain a regular sleep-wake schedule regardless of nightly variations in the quality or quantity of their sleep.
Edinger et al 2005, [35]	Standard rising time, exiting bed during extended awakenings, using the bedroom only for sleep and sex, and avoiding daytime naps.
Jungquist et al, 2010, [36]	Lie down intending to go to sleep only when sleepy, avoid any behaviour in the bed or bedroom other than sleep or sexual activity, leave the bedroom if awake for more than 15 minutes, and return to the bed only when sleepy. Keep a fixed wake time seven days a week, irrespective of the amount of sleep obtained. To prevent “clock-watching” behaviour, encourage patients to leave the bedroom as soon as they feel “clearly awake” or experience annoyance and irritation over the fact that they are awake.
Lami et al, 2018, [37]	Reinforce the relationship between sleep and bed but no further details provided.
Latocha et al, 2023, [38]	N/R
Martínez et al, 2014, [18]	N/R
McCrae et al, 2019, [39]	Do not use your bed or bedroom for anything (anytime) but sleep and sex. If you do not fall asleep within 15–20 minutes, leave the bed and do something non-stimulating in another room. Return to bed only when sleepy. If you do not fall asleep within 20 minutes upon returning to bed, repeat this instruction as needed. Avoid napping.
McCurry et al, 2021, [7]	“Behavioral/scheduling strategies” not called stimulus control: Reinforcing the associations between bed/bedroom, bedtime, and sleep - Do not use the bed/bedroom for non-sleeping activities - Allow at least one hour to unwind before bedtime - Go to bed only when sleepy - Get out of bed at the same time every morning - Avoid prolonged daytime naps or napping after 3:00 pm - Get out of bed when unable to sleep within 15–20 min
Prados et al, 2020, [10]	N/R
Smith et al, 2015, [40]	Lie down intending to go to sleep only when sleepy, avoid any behaviour in the bed or bedroom other than sleep or sexual activity, leave the bedroom if awake for more than 15 minutes, and return to the bed only when sleepy. Keep a fixed wake time seven days a week, irrespective of the amount of sleep obtained. To prevent “clock-watching” behaviour, encourage patients to leave the bedroom as soon as they feel “clearly awake” or experience annoyance and irritation over the fact that they are awake.
Vitiello et al, 2013, [41]	N/R
Wiklund et al, 2022, [42]	Participants were told to use the bedroom only for sleep and sex. That is, activities such as watching television, reading, and social media consumption were not to be conducted in the bedroom. In addition, the participants were told to go to bed only when sleepy and to get out of bed and leave the bedroom when unable to sleep. Similarly, the participants were told not to sleep in places other than the bedroom or outside the prescribed bedtimes. If stimulus control is implemented, it will restrict time in bed, similar to sleep restriction.

Abbreviations: N/R, not reported

There is variation in what form of cognitive therapy was delivered, and what this was called, and one study chose not to include this component to keep their intervention brief [42]. Interestingly, one study stated they included cognitive therapy but delivered some content within this component that other authors described under sleep education [35]. Due to lack of detail on the content of this study’s cognitive component’s, it is unclear if other cognitive restructuring content was delivered. Constructive worry and mindfulness was a frequently adopted technique but there was much variation in the prescription of same (see Table 8). Similarly, the definition of cognition and other factors such as sleep hygiene varied considerably between trials with implications for the associated treatment strategies.

**Table 8:** Cognitive component delivered in study.

Study	Description
Currie et al, 2000, [34]	Designed to help the participants explore how their thoughts, particularly their attitudes and beliefs towards sleep, affect their sleep behaviours and emotions. Participants were guided through cognitive restructuring procedures focused on reducing negative self-talk and replacing maladaptive thoughts about sleep with more adaptive substitutes. General techniques for managing presleep stress were also introduced.
Edinger et al 2005, [35]	Cognitive therapy module on audiocassette designed to correct common misconceptions about sleep needs and the effects of aging, circadian rhythms, and sleep loss and sleep/wake functioning.
Jungquist et al, 2010, [36]	Cognitive restructuring for insomnia focuses upon catastrophic thinking and the belief that poor sleep is likely to have devastating consequences. While psychoeducation may also address these kinds of issues, another ingredient of cognitive restructuring lies not in disabusing the patient of erroneous beliefs, but rather in having them discover that their estimates are not necessarily factual. When undertaking this exercise with a patient, it needs to be introduced in a considerate way, one that avoids any hint that the therapist is being pedantic, patronizing, or condescending. There are essentially 8 steps to the process. Setting the stage for cognitive restructuring Calculating how long the patient has had insomnia (in days) Identify and record between 3 and 10 catastrophic thoughts Assess the patient’s probability estimates Determine the actual frequency of occurrence of the anticipated “catastrophes” Discuss the mismatch between the patient’s estimates and the probability of catastrophic outcome Create a countering mantra to the catastrophic thoughts On review, address remaining issues regarding intrusive thoughts and negative sleep beliefs
Lami et al, 2018, [37]	Identification of dysfunctional thoughts/attitudes related to sleep (e.g., causes and consequences of sleep, sleep habits) and pain. Strategies to replace dysfunctional thoughts/attitudes for more adaptive ones–cognitive restructuring
Latocha et al, 2023, [38]	Cognitions affecting sleep and introduction to worry time
Martínez et al, 2014, [18]	Cognitive Therapy to change negative thoughts about insomnia through verbal discussion and behavioural experiments.
McCrae et al, 2019, [39]	Cognitive Therapy–monitoring automatic thoughts, instruction provided on recognising thought patterns and emotional reactions that interfere with getting good sleep (*I will never sleep again*). Cognitive Therapy–Challenging/replacing dysfunctional thoughts–instruction was provided on challenging the validity of sleep interfering thoughts and then replacing them sleep conducive ones (*there are things I can do to help my sleep*). Cognitive Therapy–practical recommendations–establish cognitive restructuring techniques (reappraisal, reattribution and decatastrophising) were taught.
McCurry et al, 2021, [7]	Constructive worry and mindfulness techniques to reduce hyperarousal at night, along with education about age related sleep changes and realistic sleep expectations. Keep realistic expectations, avoid blaming insomnia for all daytime misfortunes, revise misconceptions about the causes of insomnia, do not catastrophise after a poor night’s sleep, avoid placing too much emphasis on sleep, develop tolerance to the effects of sleep loss.
Prados et al, 2020, [10]	Cognitive Therapy—Analyse the relationships between thoughts and, feelings and behaviours related to pain and sleep (wrong implications and habits about sleep) and their relationships with activity avoidance and mood state. Challenge negative thoughts and dysfunctional beliefs about pain and sleep (strategies for modifying catastrophic appraisals of pain and sleep and replacing dysfunctional beliefs with more functional ones).
Smith et al, 2015, [40]	Cognitive restructuring for insomnia focuses upon catastrophic thinking and the belief that poor sleep is likely to have devastating consequences. While psychoeducation may also address these kinds of issues, another ingredient of cognitive restructuring lies not in disabusing the patient of erroneous beliefs, but rather in having them discover that their estimates are not necessarily factual. When undertaking this exercise with a patient, it needs to be introduced in a considerate way, one that avoids any hint that the therapist is being pedantic, patronizing, or condescending. There are essentially 8 steps to the process. Setting the stage for cognitive restructuring Calculating how long the patient has had insomnia (in days) Identify and record between 3 and 10 catastrophic thoughts Assess the patient’s probability estimates Determine the actual frequency of occurrence of the anticipated “catastrophes” Discuss the mismatch between the patient’s estimates and the probability of catastrophic outcome Create a countering mantra to the catastrophic thoughts On review, address remaining issues regarding intrusive thoughts and negative sleep beliefs
Vitiello et al, 2013, [41]	Cognitive restructuring
Wiklund et al, 2022, [42]	Component not delivered

#### CBT-I format

Table 9 describes the specific format of CBT-I in each study. All studies delivered CBT-I interventions over five to nine weeks. Ten of the 12 studies described weekly sessions, one study provided six sessions over eight weeks [7] and one study failed to include this detail [40]. Limited detail was provided across the studies about where the intervention was delivered. One study stated CBT-I was delivered remotely via telephone consultations [7] and another delivered content online but failed to detail if this was pre-recorded content or delivered live [42]. It is uncertain if the remaining ten interventions were carried out face to face, but we assumed that they were. Of the eight studies who stated, six studies had group sessions [10, 18, 34, 37, 38, 41]. Group sizes ranged from 5–12 participants, although the most recent study failed to describe the group size [38]. Of the six studies using group interventions, three studies delivered combined CBT-I and CBT-P [10, 37, 41] and three studies delivered CBT-I alone [18, 34, 38]. All combined CBT-I and CBT-P interventions were carried out in groups of either 5–7 or 5–12 [10, 37, 41]. The length of time of each session varied from 15–90 minutes for individual sessions and 90–120 minutes for group sessions. One study failed to record the session length [42].

**Table 9:** Format of CBT-I.

Study	Group size	Delivery	Total time	CBT-I/IP/C
Currie et al, 2000, [34]	5–7	120 mins x 7	840m	CBT-I
Lami et al, 2018, [37]	5–7	90 mins x 9	810m	CBT- IP
Latocha et al, 2023, [38]	N/R	120 mins x 6	720m	CBT-I
Martínez et al, 2014, [18]	5–6	90 mins x 6	540m	CBT-I
Prados et al, 2020, [10]	5–7	90 mins x 9	810m	CBT-C
Vitiello et al, 2013, [41]	5–12	90 mins x 6	540m	CBT-PI
Edinger et al 2005, [35]	1	1^st^ session 45-60mins	120m –210m	CBT-I
		2^nd^– 6^th^ 15 – 30mins		
Jungquist et al, 2010, [36]	1	30 – 90mins x 8	285-435m	CBT-I
McCrae et al, 2019, [39]	1	50 mins x 8	400m	CBT-I
McCurry et al, 2021, [7]	1	20–30 mins x 6	120-180m	CBT-I
Smith et al, 2015, [40]	1	45 mins x 8	360m	CBT-I
Wiklund et al, 2022, [42]	1	Mins N/R x 5	N/R	CBT-I

CBT-I indicates Cognitive Behavioural Therapy for Insomnia; CBT-IP/CBT-PI/CBT-C, Cognitive Behavioural Therapy for Insomnia and Pain; Mins/m, minutes; N/R, not reported

The professional background of those delivering the intervention was usually stated. CBT-I was carried out by Clinical Psychologists in three studies [7, 35, 41], pre/post-doctoral students in clinical psychology in three studies [34, 39, 40], therapists in two studies [18, 37], masters/PhD trained nurses in three studies [7, 36, 38], post-doctoral psychology fellows/faculty in clinical psychology in one study [40], post-doctoral researchers in clinical psychology in one study [10], masters students in psychology or senior psychologists in one study [42] social workers in one study [7] and family counsellors in one study [41]. Most studies provided no detail on the specific expertise in CBT-I, or the training undertaken to deliver CBT-I.

## Discussion

### Summary of evidence

This review aimed to map the content and format of CBT-I in RCTs in patients with comorbid disordered sleep and persistent musculoskeletal pain. The findings of this review would suggest there was considerable consistency in the components of CBT-I delivered in clinical trials along with certain aspects of delivery (e.g. frequency and number of sessions). However, some aspects were either not reported (e.g., content of components) or not consistent (e.g., use of terminology). Lack of detail provided across the studies impacted the ability to chart and compare the interventions between RCTs.

### Content

This review found there was considerable consistency in two core components of CBT-I delivered across the included studies; sleep restriction and stimulus control. These findings are consistent with the core components used in other programmes assessing night time sleep disturbance and day time functioning in people with chronic insomnia [47] and sleep quality, daytime functioning, quality of life and fatigue in people with cancer [48]. Although, a cognitive component is typically also included in these studies.

Morin et al, 2006 describe CBT-I as any combination of the behavioral (e.g., stimulus control, sleep restriction, relaxation) and cognitive procedures [49] and this aligns, for the most part, with the findings of our study but it also describes a key feature of CBT-I. The flexibility to choose which components to deliver to the target cohort may be what is reflected here in the variation in additional components delivered alongside the core components. Some evidence suggest additional components might include a cognitive component [49], sleep hygiene [50], relaxation techniques [51] or sleep education [52]. Perhaps in patients with comorbid disordered sleep and persistent pain, the choice of relaxation techniques would be the additional component of choice as it has the benefit of decreasing arousal and stress, both of which are keys factors in managing persistent pain [53].

Limited detail provided on the content delivered in each component mirrors other studies which report similar issues with poor reporting of content of CBT-I components [54]. As a result we do not really know the precise content delivered in each component and there may be considerable variation. When detail was provided there was an abundance of information [36, 40] and usually involved referencing detailed treatment manuals, findings supported by other studies [55].

Persistent pain and insomnia are two of the most common complaints seen in General Practice and primary care [53, 56] yet demand for professionals trained to deliver CBT-I exceeds supply [53]. With appropriate training, this is an approach that can be adopted by a range of professionals. This review found that many different professionals delivered CBT-I, but limited detail was provided on what training was undertaken. Many of the skills required to deliver CBT-I are already inherent in the training and work of many professionals including General Practitioners, nurses and physiotherapists [57]. These professionals are progressively delivering psychologically informed treatments [57–59] and are more accessible professionals to this patient group than clinical psychologists who were most often used in the RCTs included in this review.

Taking into consideration the findings of recent studies, training might come in the form of an in-depth workshop and would benefit from including some experiential learning such as supervision [55, 57, 58]. The review found CBT-I was delivered by a number of professions with variable experience and specific CBT-I training. While it may be easier to achieve confidence in applying stimulus control and sleep restriction, there may be a perceived barrier to delivering a cognitive component. Front line therapists deliver elements of cognitive restructuring in relation to pain e.g. advice on catastrophising and pacing, but there may be a hesitancy when content is applied to sleep [57]. A study where GP’s and nurses delivered CBT-I to chronic insomnia patients found the practitioners felt the need for more extensive training having received two sessions of two hours duration [59]. The need for more training [59] and clinical supervision [57] on the cognitive component was identified with some professionals asking for this component to be removed completely. However, it is understood that the cognitive component of CBT-I leads to changes in dysfunctional beliefs about sleep which is predictive of positive outcomes [60, 61]. A stepped approach to CBT-I has been suggested [62] which would allow professionals such as GP’s or physiotherapists to deliver basic elements aligned with CBT-I, with a clear pathway for when to refer on to more skilled professionals, this might encourage professionals to use this treatment clinically.

### Format

This review found CBT-I primarily took the form of individual and group face to face sessions, one study delivering individual sessions of telephone CBT-I [7] and another study delivering online sessions [42]. These findings closely aligned with two studies carried out on patients with chronic insomnia [63, 64], a study on cancer patients [65] and a study on traumatic brain injury patients [54] in relation to the mix of individual and group sessions and the delivery method.

The flexibility of format offers clinicians options when addressing some of the patient barriers faced when delivering CBT-I [53] such as limited time, available clinic hours, travel logistics and waiting times. However, a study exploring patients perspective of CBT-I format found that patients reported face to face delivery as superior to eCBT-I but they did feel that there was a place for eCBT-I for relapse management [66]. This study recommended that the patients perspective is explored prior to commencing treatment to ensure the format of CBT-I meets the need of each patient. Recent changes to healthcare delivery due to the pandemic has required services to become generally more flexible [67].

The range of delivery times for each intervention in this study is of note. It is difficult to compare the content of an individual course of CBT-I delivered over a total period of 120 minutes, a group intervention of 840 minutes delivered to a group of 5–12 patients and a group where no detail was provided on the session length. The variation in delivery time is not isolated to this cohort, numerous studies report similar findings for cancer patients [65, 68], traumatic brain injury patients [54] and insomnia [51]. This may be in part due to the option to tailor CBT-I components. Of the studies that delivered combined CBT-I and CBT-P, all had session lengths of 90 minutes, however the number of sessions delivered varied. Offering six sessions [41] instead of nine sessions [10, 37], translates to a difference of 260 minutes in total delivery time and is likely to lead to a reduction in the amount of CBT-I content delivered. The impact fewer sessions has on the amount of CBT-I content delivered, and the subsequent impact this may have on treatment outcomes for this combined approach warrants exploration.

There appears to be consistency in the amount of sessions delivered in this review however, this contrasts to the four individual biweekly sessions that have been shown to be the most effective [69]. In addition a study delivering CBT-I to chronic insomnia patients found a week by week improvement in TST [47]. It is yet to be determined if the variation in amount of sessions in this review from 5 to 9 weeks could impact the intervention and this would benefit from further research.

### Strengths and limitations

The strengths of this review include the prospective protocol registration, following the PRISMA ScR guidance, searching comprehensively across nine databases and the inclusion of PEDro scores. The structured approach to data analysis offered by using the TIDieR Checklist is also a strength of this study. This review focussed on RCTs which could limit the findings, however we are reassured as a systematic review of CBT-I with a different focus published in 2021, included the same ten studies published at this time [8]. Studies were limited to English or those translated to English. There may be gender bias in this review due to the largely female sample in the associated studies (82%), although this may reflects the dominance of female patients with FM. Lack of detail provided by the studies for the TIDieR items make it difficult to compare each intervention, this limitation is compounded by the inconsistency in the terminology used across the studies.

### Clinical implications

If looking to deliver CBT-I clinically for this cohort, this review has found that current CBT-I programmes always deliver sleep restriction and stimulus control and frequently deliver a cognitive component, sleep hygiene, sleep education, a form ofs relaxation and relapse prevention. Further research in defining the content of each core component along with the terminology and the creation of guidelines would help to address the lack of details in the studies included.

Fidelity was assessed in ten studies with some studies providing details of thorough strategies to ensure fidelity and adherence. In a clinical environment it does not appear feasible nor realistic to carry out the breadth of strategies described, although ensuring all therapists have received suitable training and follow a treatment manual may be more feasible. The use of adherence questionnaires could be easily implemented but the honesty of the responses might be compromised due to limited anonymity in the clinical environment.

### Future research

This scoping review highlights the need to define the content of each core component along with the terminology, perhaps with a view to creating a CBT-I guideline. Exploration into component selection for specific conditions such as OA or FM would be helpful. Further exploration into how much training therapists would need to feel competent to carry out CBT-I and the associated delivery and training costs would be beneficial.

## Conclusion

These findings demonstrate considerable consistency in both the components of CBT-I delivered in clinical trials along with the number of sessions. The frequency of sessions was also consistent where almost all studies held weekly sessions. However, some aspects were either not reported (e.g., content of components) or not consistent (e.g., use of terminology). CBT-I was delivered both individually and in groups. Lack of detail provided across the studies impacted the ability to compare the interventions. Greater consistency, and more detailed reporting regarding who delivered the intervention, the training provided, and the specific content of CBT-I components would be of great benefit.

## Supporting information

S1 Fig Search strategy. (TIF)

S2 Fig Preferred Reporting Items for Systematic Reviews and Meta-Analyses (PRISMA) guidelines. (TIF)

S3 Fig Preferred Reporting Items for Systematic Reviews and Meta-Analyses extension for Scoping Reviews (PRISMA-ScR) checklist. (TIF)
