# Supplementary figures and images for: How is cognitive behavioural therapy for insomnia delivered to adults with comorbid persistent musculoskeletal pain and disordered sleep? A scoping review
Source: PLoS One. 2024 Jul 18;19(7):e0305931. doi: 10.1371/journal.pone.0305931 (PMC11257322; doi:10.1371/journal.pone.0305931)

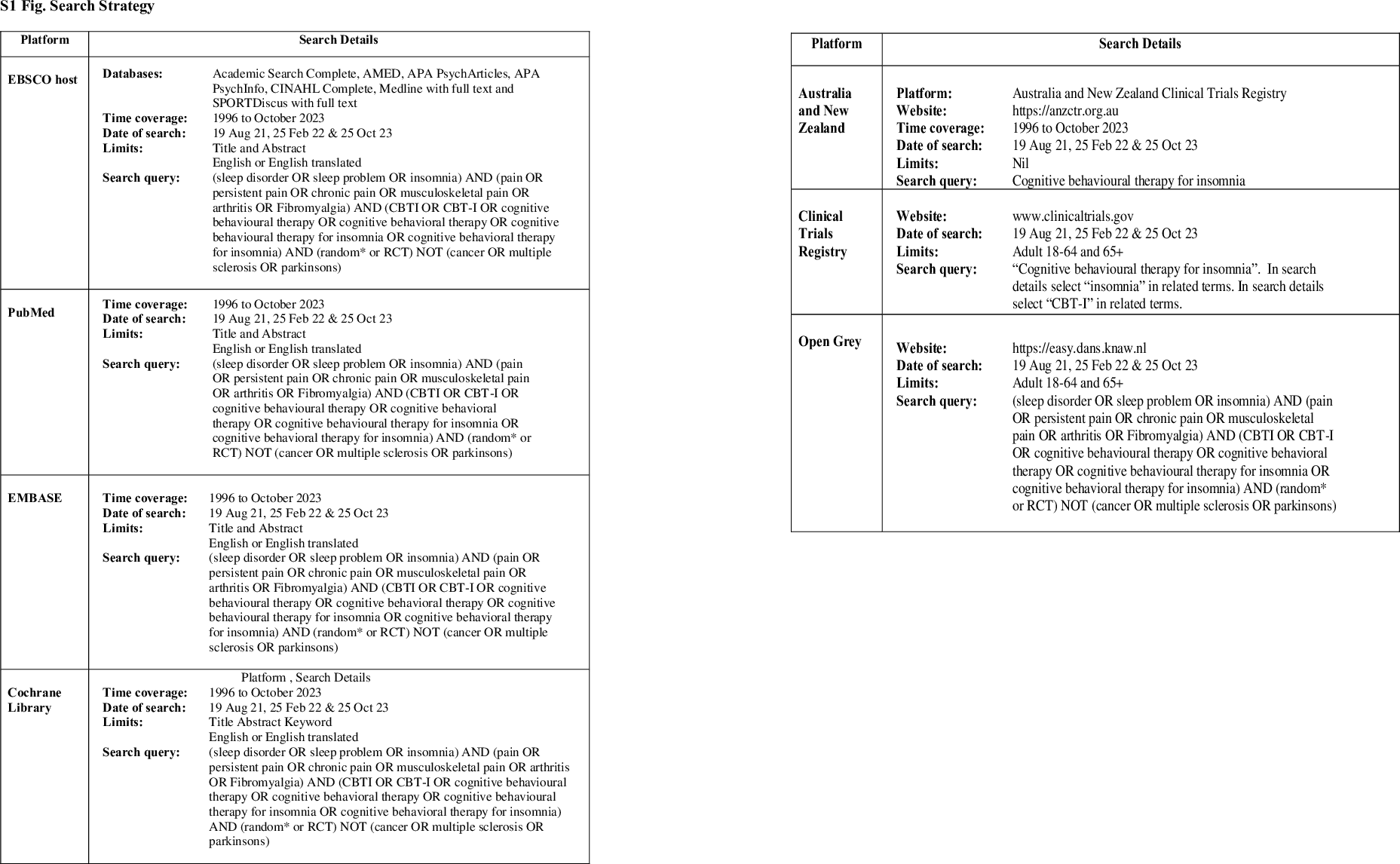

Supplement: S1 Fig — (TIF) [file pone.0305931.s001.tif]

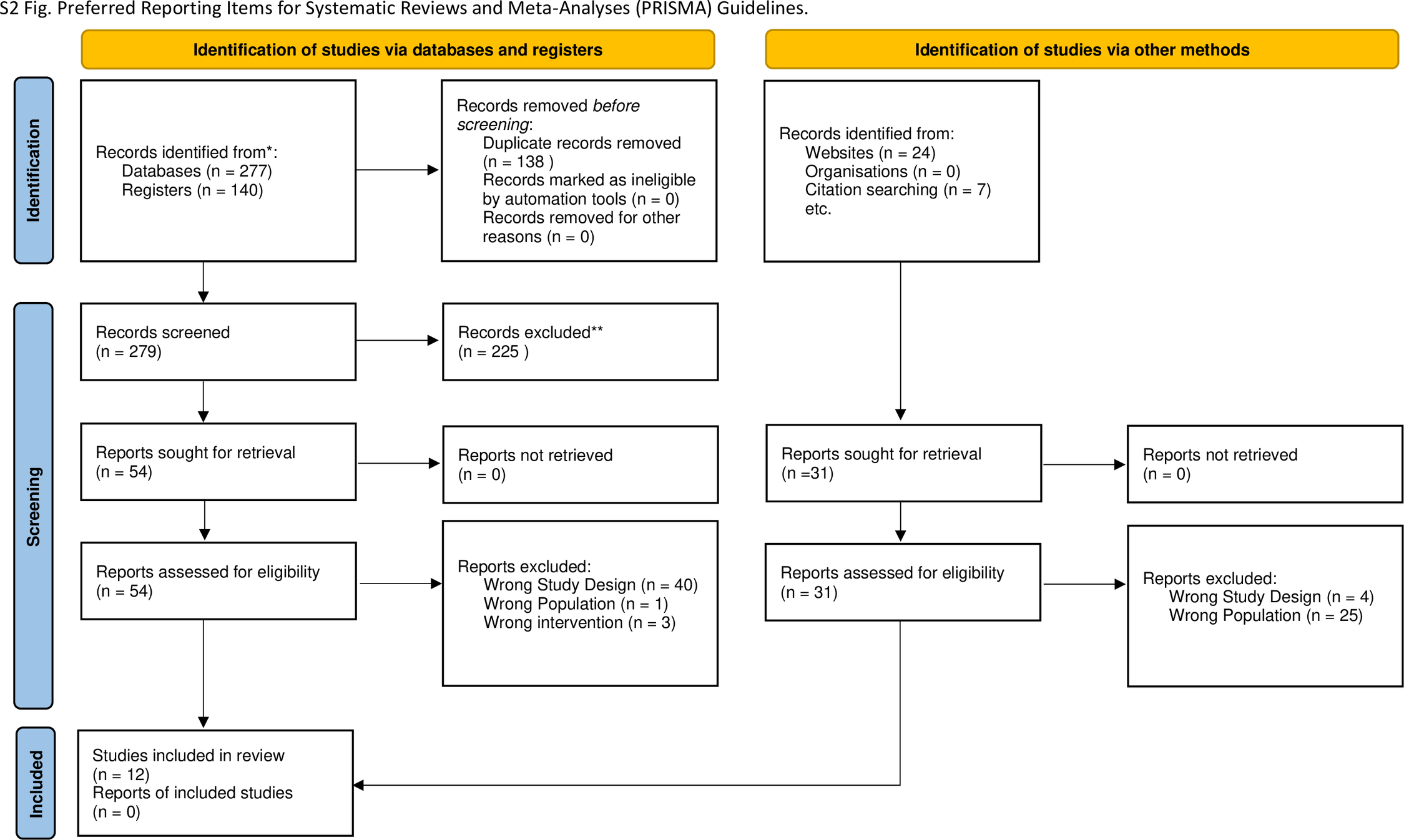

Supplement: S2 Fig — (TIF) [file pone.0305931.s002.tif]

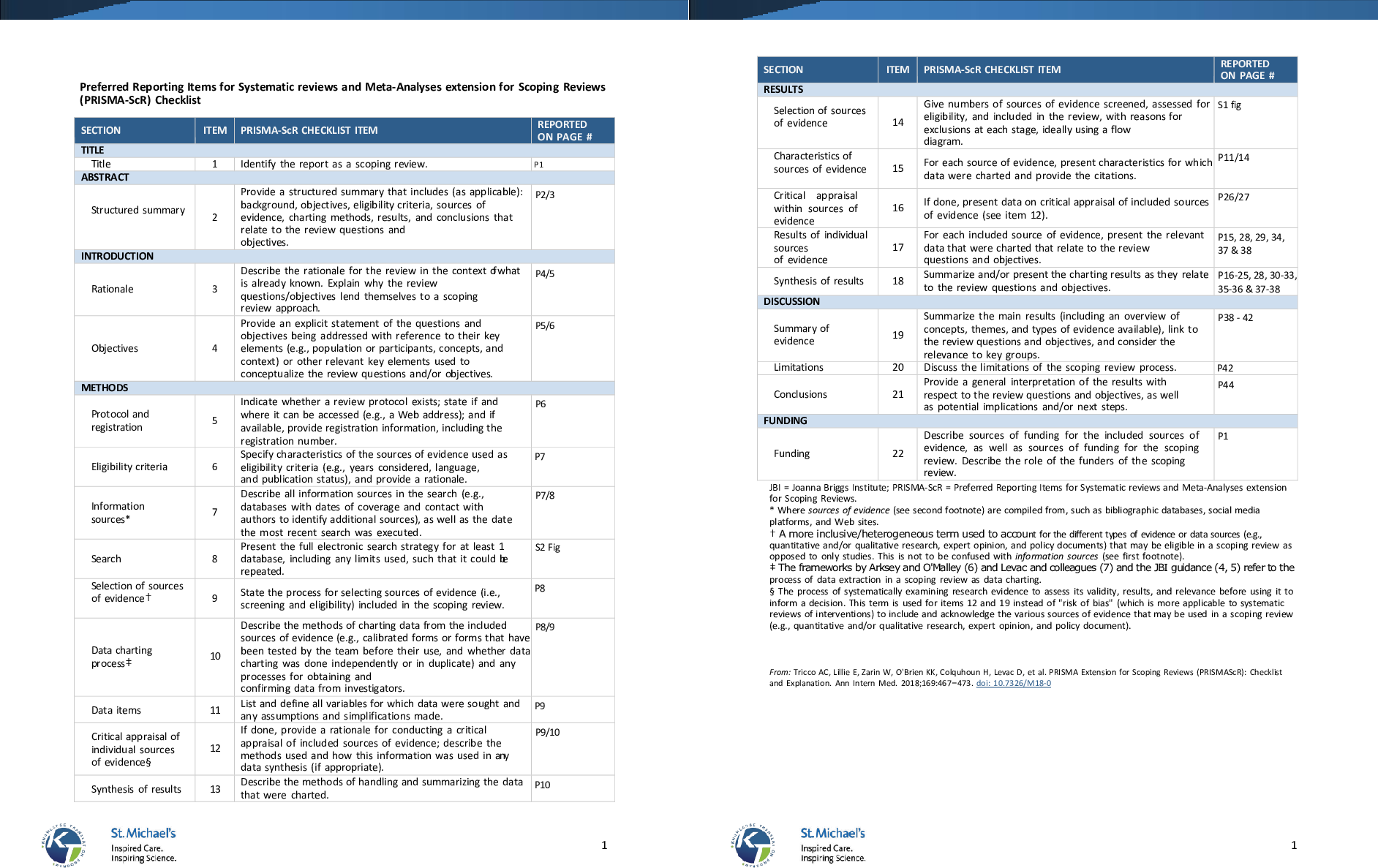

Supplement: S3 Fig — (TIF) [file pone.0305931.s003.tif]
